# Supplementary material for: Fear perception as a function of hemisphere- and time-specific dynamics in the medial temporal lobes
Source: Commun Biol. 2025 Jul 25;8:1105. doi: 10.1038/s42003-025-08542-6 (PMC12297512; doi:10.1038/s42003-025-08542-6)
Supplement: Supplementary file 2 — Supplementary Information [file 42003_2025_8542_MOESM2_ESM.pdf]

Supplementary Information for

Fear perception as a function of hemisphere- and time-specific dynamics in the medial temporal lobes

Enya M. Weidner, Lea Marie Reisch, Malena Mielke, Christian G. Bien, Johanna Kissler

Correspondence to: Enya Weidner
Email: enya.weidner@uni-bielefeld.de

S1 Linear mixed model coefficients: behavioral data

Supplementary Table 1. Mean values of stimulus ratings per group.

	Arousal			Valence		
	Fearful	Neutral	Mean	Fearful	Neutral	Mean
lTLR *M* (*SD*)	4.250 (1.479)	2.922 (1.012)	3.586 (1.419)	2.950 (0.916)	3.711 (0.943)	3.331 (0.994)
HC *M* (*SD*)	4.011 (1.411)	2.917 (1.083)	3.464 (1.358)	2.628 (0.570)	3.722 (0.437)	3.175 (0.747)
rTLR *M* (*SD*)	4.176 (1.277)	3.141 (0.679)	3.659 (1.136)	2.818 (0.697)	3.718 (0.667)	3.268 (0.812)
Mean *M* (*SD*)	4.145 (1.370)	2.991 (0.933)	3.569 (1.303)	2.798 (0.741)	3.717 (0.700)	3.258 (0.853)

Note. Ratings were given on a 7-point Likert scale, 1 being most negative/least arousing. Abbreviations: HC = healthy controls, lTLR = left temporal lobe resection, M = mean, rTLR = right temporal lobe resection, SD = standard deviation.

Supplementary Table 2. Model summary statistics of stimulus ratings.

Coefficient	β	*SE*	*t*	*p*
Arousal				
Intercept (Grand Mean)	**3.570**	**0.116**	**30.846**	**< 2e-^{16}**
Group (lTLR)	0.089	0.165	0.540	.590
Group (rTLR)	-0.106	0.163	-0.649	.518
Emotion	**0.576**	**0.116**	**4.980**	**2.67e^{-06}**
Group (lTLR) × Emotion	-0.059	0.163	-0.355	.724
Group (rTLR) × Emotion	-0.029	0.163	-0.178	.859
Valence				
Intercept (Grand Mean)	3.258	0.071	46.037	**< 2e^{-16}**
Group (lTLR)	0.010	0.101	0.098	.922
Group (rTLR)	-0.083	0.100	-0.831	.408
Emotion	**-0.459**	**0.071**	**-6.490**	**3.33e^{-07}**
Group (lTLR) × Emotion	0.009	0.101	0.092	.927
Group (rTLR) × Emotion	-0.088	0.100	-0.883	.379

Note. *T*-statistics are evaluated for significance. A positive coefficient indicates higher ratings in the test group than in the group mean. Effects with $p \leq .05$ are marked in bold. Abbreviations: HC = healthy controls, lTLR = left temporal lobe resection, rTLR = right temporal lobe resection, SE = standard error of mean.

Supplementary Table 3. Mean values of recognition scores per group.

	DI			Bias		
	Fearful	Neutral	Mean	Fearful	Neutral	Mean
lTLR *M* (*SD*)	0.209 (0.204)	0.250 (0.176)	0.230 (0.189)	0.461 (0.210)	0.357 (0.172)	0.409 (0.196)
HC *M* (*SD*)	0.196 (0.190)	0.257 (0.202)	0.227 (0.206)	0.510 (0.175)	0.378 (0.141)	0.444 (0.167)
rTLR *M* (*SD*)	0.118 (0.108)	0.145 (0.185)	0.132 (0.150)	0.490 (0.174)	0.347 (0.226)	0.419 (0.212)
Mean *M* (*SD*)	0.174 (0.179)	0.217 (0.195)	0.196 (0.188)	0.487 (0.183)	0.361 (0.180)	0.424 (0.192)

Note. Recognition scores are reported as the corrected discrimination index (Hits - False alarms) according to the two-high threshold model.[1] The response bias was calculated as false alarms divided by 1-recognition performance. Abbreviations: DI = discrimination index, HC = healthy controls, lTLR = left temporal lobe resection, M = mean, rTLR = right temporal lobe resection, SD = standard deviation.

Supplementary Table 4. Model summary statistics of memory performance.

Coefficient	β	*SE*	*t*	*p*
DI				
Intercept (Grand Mean)	**0.197**	**0.018**	**10.981**	**< $2e^{-16}$**
Group (lTLR)	0.032	0.025	1.282	.203
Group (rTLR)	**-0.065**	**0.026**	**-2.549**	**.012**
Emotion	-0.021	0.018	-1.224	.224
Group (lTLR) × Emotion	0.002	0.025	0.076	.940
Group (rTLR) × Emotion	0.009	0.025	0.334	.739
Recognition bias				
Intercept (Grand Mean)	**0.429**	**0.017**	**24.087**	**< $2e^{-16}$**
Group (lTLR)	-0.020	0.025	-0.816	.417
Group (rTLR)	-0.010	0.025	-0.406	.686
Emotion	**0.644**	**0.018**	**3.620**	**.0005**
Group (lTLR) × Emotion	-0.012	0.025	-0.497	.620
Group (rTLR) × Emotion	0.007	0.025	0.288	.774

Note. *T*-statistics are evaluated for significance. A positive coefficient indicates higher ratings in the test group than in the group mean. Effects with $P \leq .05$ are marked in bold. Abbreviations: DI = discrimination index, HC = healthy controls, lTLR = left temporal lobe resection, rTLR = right temporal lobe resection, SE = standard error of mean.

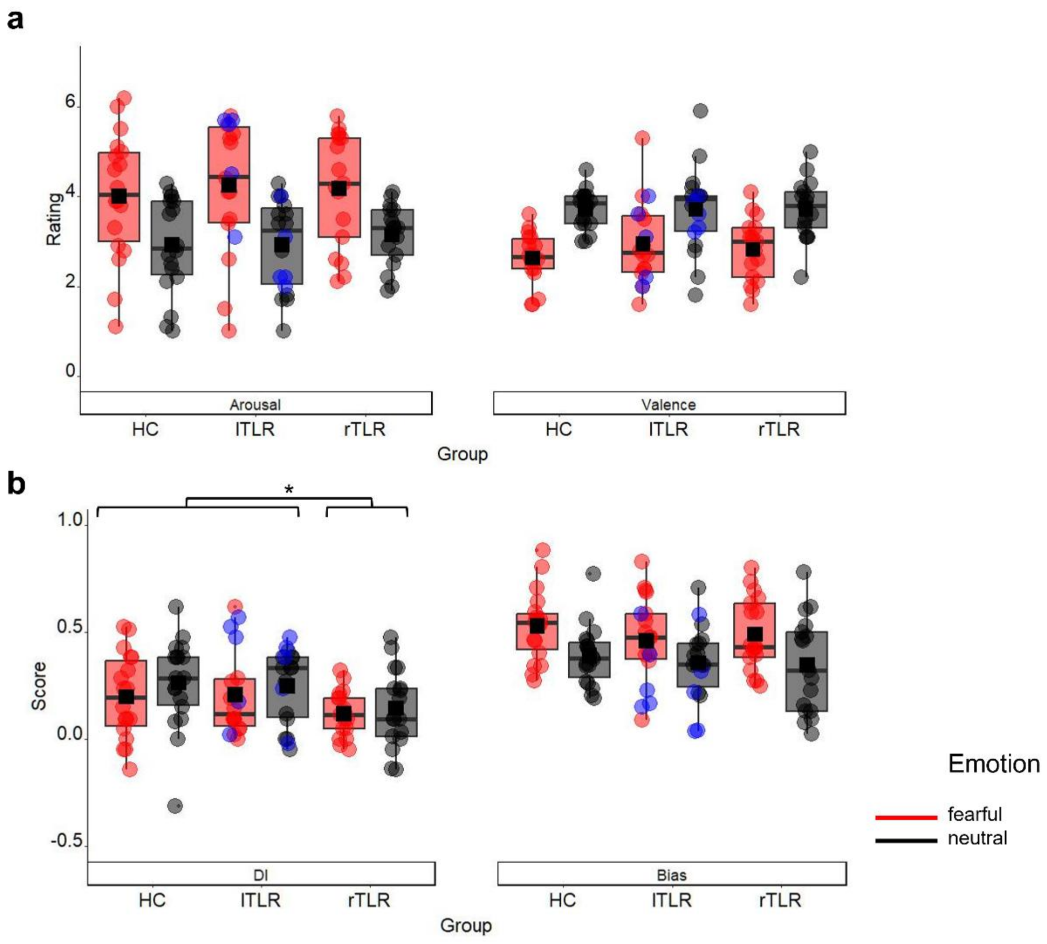

Supplementary Figure 1. Stimulus ratings and memory performance. Depicted are boxplots that show the distribution of **a** stimulus ratings and **b** mean memory performance scores. Whiskers indicate the interquartile range. The bold horizontal line indicates the distribution median. Dots indicate single-subject values (n = 18 per group). Black squares indicate the distribution mean. lTLR patients with apical resections (spared hippocampus, n = 5) are marked in blue. Brackets mark significant comparisons (*$p \leq .05$). Abbreviations: DI = discrimination index, HC = healthy controls, lTLR = left temporal lobe resection, rTLR = right temporal lobe resection.

S2 Linear mixed model coefficients: EEG

Supplementary Table 5. Model summary statistics of the ERPs.

Coefficient	β	*SE*	*t*	*df*	*p*
Intercept (Grand Mean)	**1.777**	**0.250**	**7.109**	**51**	**$3.680e^{-09}$**
Emotion	**-0.156**	**0.016**	**-9.935**	**115700**	**< 2e-16**
Group (lTLR)	-0.214	0.354	-0.606	51	.557
Group (rTLR)	0.077	0.354	0.217	51	.839
ERP (LPP)	**-1.237**	**0.291**	**-4.252**	**51**	**$9.070e^{-05}$**
ERP (N1)	**-2.782**	**0.325**	**-8.552**	**51**	**$2.050e^{-11}$**
ERP (P1)	**0.795**	**0.197**	**4.029**	**51**	**$1.880e^{-04}$**
Hemisphere	**-0.167**	**0.016**	**-10.694**	**115700**	**$< 2e^{-16}$**
Emotion × Group (lTLR)	-0.027	0.022	-1.200	115700	.237
Emotion × Group (rTLR)	-0.015	0.022	-0.679	115700	.508
Emotion × ERP (LPP)	**0.349**	**0.027**	**12.870**	**115700**	**$< 2e^{-16}$**
Emotion × ERP (N1)	**-0.298**	**0.027**	**-10.977**	**115700**	**$< 2e^{-16}$**
Emotion × ERP (P1)	**0.203**	**0.027**	**7.470**	**115700**	**$< 2e^{-16}$**
Group (lTLR) × ERP (LPP)	0.776	0.412	1.887	51	.073
Group (rTLR) × ERP(LPP)	-0.419	0.411	-1.019	51	.295
Group (lTLR) × ERP (N1)	-0.108	0.460	-0.234	51	.809
Group (rTLR) × ERP (N1)	-0.397	0.460	-0.863	51	.396
Group (lTLR) × ERP (P1)	-0.057	0.279	-0.205	51	.853
Group (rTLR) × ERP (P1)	0.053	0.279	0.190	51	.853
Emotion × Hemisphere	-0.003	0.016	-0.205	115700	.854
Group (lTLR) × Hemisphere	**0.088**	**0.022**	**3.932**	**115700**	**$8.430e^{-05}$**
Group (rTLR) × Hemisphere	**-0.167**	**0.022**	**-7.669**	**115700**	**$1.740e^{-14}$**
ERP (LPP) × Hemisphere	**0.122**	**0.027**	**4.500**	**115700**	**$6.790e^{-06}$**
ERP (N1) × Hemisphere	**0.528**	**0.027**	**19.502**	**115700**	**$< 2e^{-16}$**
ERP (P1) × Hemisphere	**-0.274**	**0.027**	**-10.108**	**115700**	**$< 2e^{-16}$**
Emotion × Group (lTLR) × ERP (LPP)	**0.094**	**0.039**	**2.441**	**115700**	**.017**
Emotion × Group (rTLR) × ERP (LPP)	-0.019	0.038	-0.495	115700	.621
Emotion × Group (lTLR) × ERP (N1)	**-0.081**	**0.039**	**-2.103**	**115700**	**.040**
Emotion × Group (rTLR) × ERP (N1)	0.042	0.038	1.122	115700	.254
Emotion × Group (lTLR) × ERP (P1)	0.039	0.039	1.022	115700	.304
Emotion × Group (rTLR) × ERP (P1)	**-0.081**	**0.038**	**-2.138**	**115700**	**.025**
Emotion × Group (lTLR) × Hemisphere	-0.034	0.022	-1.520	115700	.132
Emotion × Group (rTLR) × Hemisphere	0.003	0.022	0.122	115700	.917
Emotion × ERP (LPP) × Hemisphere	-0.006	0.027	-0.229	115700	.805
Emotion × ERP (N1) × Hemisphere	-0.001	0.027	-0.049	115700	.959
Emotion × ERP (P1) × Hemisphere	0.000	0.027	0.002	115700	.997
Group (lTLR) × ERP (LPP) × Hemisphere	**-0.173**	**0.039**	**-4.483**	**115700**	**$7.380e^{-06}$**
Group (rTLR) × ERP (LPP) × Hemisphere	**0.194**	**0.038**	**5.118**	**115700**	**$3.090e^{-07}$**
Group (lTLR) × ERP (N1) × Hemisphere	-0.067	0.039	-1.730	115700	.074
Group (rTLR) × ERP (N1) × Hemisphere	**-0.174**	**0.038**	**-4.611**	**115700**	**$4.010e^{-06}$**
Group (lTLR) × ERP (P1) × Hemisphere	0.013	0.039	0.342	115700	.729
Group (rTLR) × ERP (P1) × Hemisphere	**0.199**	**0.038**	**5.274**	**115700**	**$1.340e^{-07}$**
Emotion × Group (lTLR) × ERP (LPP) × Hemisphere	0.010	0.039	0.268	115700	.794
Emotion × Group (rTLR) × ERP (LPP) × Hemisphere	0.009	0.038	0.245	115700	.786
Emotion × Group (lTLR) × ERP (N1) × Hemisphere	-0.020	0.039	-0.528	115700	.611
Emotion × Group (rTLR) × ERP (N1) × Hemisphere	-0.025	0.038	-0.670	115700	.508
Emotion × Group (lTLR) × ERP (P1) × Hemisphere	0.046	0.039	1.191	115700	.227
Emotion × Group (rTLR) × ERP (P1) × Hemisphere	0.022	0.038	0.590	115700	.534

Note. A positive coefficient indicates higher amplitudes in the test condition than group mean. For the hemisphere contrast, higher values indicate higher amplitudes over the left hemisphere. For the emotion contrast, higher values indicate higher amplitudes for fearful faces. For factors with more than two levels, multiple contrasts were calculated. Effects with $p \leq .05$ are marked in bold. Abbreviations: EPN = early posterior negativity, ERP = event-

related potential, LPP = late positive potential, HC = healthy controls, lTLR = left temporal lobe resection, rTLR = right temporal lobe resection, SE = standard error of mean.

Supplementary Table 6. Group-wise separate comparisons of estimated marginal means for fearful versus neutral faces in each ERP component.

ERP	Coefficient	β	*d*	*SE*	*CI low*	*CI up*	*t-ratio*	*df*	*p*
P1	HC	**0.260**	**0.494**	**0.110**	**0.0447**	**0.475**	**2.367**	**115719**	**.018**
	lTLR	0.119	0.185	0.110	-0.096	0.335	1.087	115721	.277
	rTLR	-0.097	-0.234	0.105	-0.3041	0.109	-0.923	115721	.356
P1 (shifted cluster)	HC	0.062	0.334	0.054	-0.043	0.167	1.160	9385	.270
	lTLR	**0.106**	**0.410**	**0.059**	**-0.008**	**1.640**	**1.816**	**9370**	**.050**
	rTLR	-0.023	-0.104	0.054	-0.125	0.085	-0.380	10180	.696
N1	HC	**-0.745**	**-1.025**	**0.110**	**-0.9605**	**-0.530**	**-6.788**	**115717**	**1.142e^{-11}**
	lTLR	**-1.122**	**-1.296**	**0.110**	**-1.3371**	**-0.906**	**-10.206**	**115717**	**1.900e^{-24}**
	rTLR	**-0.851**	**-1.231**	**0.105**	**-1.0575**	**-0.644**	**-8.066**	**115717**	**7.350e^{-16}**
EPN	HC	**-0.745**	**-1.219**	**0.110**	**-0.9598**	**-0.529**	**-6.781**	**115717**	**1.195e^{-11}**
	lTLR	**-0.977**	**-0.810**	**0.110**	**-1.1923**	**-0.762**	**-8.890**	**115717**	**6.253e^{-19}**
	rTLR	**-0.734**	**-1.039**	**0.105**	**-0.9406**	**-0.527**	**-6.957**	**115717**	**3.486e^{-12}**
LPP	HC	**0.319**	**0.604**	**0.110**	**0.1037**	**0.534**	**2.904**	**115724**	**.008**
	lTLR	**0.522**	**1.127**	**0.110**	**0.3062**	**0.737**	**4.746**	**115728**	**2.073e^{-06}**
	rTLR	**0.317**	**0.784**	**0.105**	**0.1129**	**0.526**	**3.030**	**115727**	**.006**

Note. A positive coefficient indicates higher amplitudes for fearful faces. Effects with $p \leq .05$ are marked in bold. The shifted cluster for the P1 analysis was chosen based on post-hoc evaluation of the differential topographies, in which a topographic shift of the fear-neutral differentiation was visible for lTLR patients. *P*-values are Holm corrected. Abbreviations: CI = confidence interval (95 %), EPN = early posterior negativity, ERP = event-related potential, LPP = late positive potential, HC = healthy controls, lTLR = left temporal lobe resection, rTLR = right temporal lobe resection, SE = standard error of mean, Stand. = standardized.

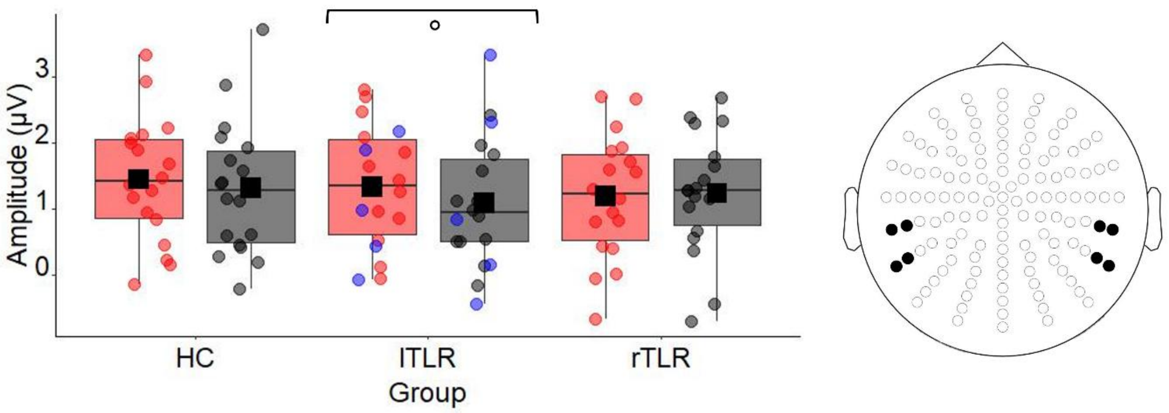

Supplementary Figure 2. Data distributions in the shifted P1 electrode cluster. Boxplots of P1 amplitudes extracted from the shifted channel clusters as marked in the topography. Data are averaged across time-points. Whiskers indicate the interquartile range. The bold horizontal line indicates the distribution median. Dots indicate single-subject values (n = 18 per group). lTLR patients with apical resections (spared hippocampus) are marked in blue (n = 5). Brackets mark significant comparisons (°$p \leq .10$). Abbreviations: HC = healthy controls, lTLR = left temporal lobe resection, rTLR = right temporal lobe resection

Supplementary Table 7. Model summary statistics and separate comparisons of the EPN.

Coefficient	β	*SE*	*t*	*df*	*p*
Intercept (Grand Mean)	**5.002**	**0.529**	**9.460**	**51**	**$8.25e^{-13}$**
Emotion	**-0.825**	**0.032**	**-12.720**	**28930**	**$< 2e^{-16}$**
Group (lTLR)	0.840	0.748	-1.104	51	.266
Group (rTLR)	-0.409	0.748	1.123	51	.240
Hemisphere	**-0.543**	**0.032**	**-16.894**	**28930**	**$< 2e^{-16}$**
Group (lTLR) × Emotion	-0.080	0.046	-1.741	28930	.084
Group (rTLR) × Emotion	0.043	0.045	0.964	28930	.355
Group (lTLR) × Hemisphere	0.314	0.032	0.119	28930	.897
Group (rTLR) × Hemisphere	**-0.386**	**0.046**	**6.863**	**28930**	**$7.22e^{-12}$**
Emotion × Hemisphere	**0.004**	**0.045**	**-8.610**	**28930**	**$< 2e^{-16}$**
Group (lTLR) × Emotion × Hemisphere	-0.070	0.046	-1.536	28930	.136
Group (rTLR) × Emotion × Hemisphere	-0.003	0.045	-0.065	28930	.933

Note. A positive coefficient indicates higher amplitudes in the test condition than group mean. For the hemisphere contrast, higher values indicate higher amplitudes over the left hemisphere. For the emotion contrast, higher values indicate higher amplitudes for fearful faces. For factors with more than two levels, multiple contrasts were calculated. Effects with $p \leq .05$ are marked in bold. Abbreviations: EPN = early posterior negativity, HC = healthy controls, lTLR = left temporal lobe resection, rTLR = right temporal lobe resection, SE = standard error of mean.

Supplementary Table 8. Summary statistics of cluster-based permutation tests and separate comparisons of GBA.

Coefficient	Time (ms)		Freq (Hz)			Summed *t*		Cluster *p*
Emotion	**85-195**		**70-85**			**-627.929**		**.001**
Group (lTLR)	**90-340**		**35-75**			**-2721.000**		**.001**
	**620-800**		**70-85**			**-2295.500**		**.007**
**Group (rTLR)**	**150-390**		**35-50**			**-2844.100**		**.001**
	**85-385**		**35-90**			**13330.000**		**.001**
Group (lTLR) × Emotion						*No clusters*		
Group (rTLR) × Emotion	**95-300**		**60-80**			**-1421.000**		**.0002**
Post-hoc tests: Group (rTLR) × Emotion								
Coefficient	β	*d*	*SE*	*CI low*	*CI up*	*t*-ratio	*df*	*p*
HC: fear/neutral	0.014	0.351	0.010	-0.005	0.033	1.489	14115	.137
lTLR: fear/neutral	0.008	0.228	0.010	-0.011	0.027	0.872	14115	.383
rTLR: fear/neutral	**-0.024**	**-0.658**	**0.009**	**-0.042**	**-0.006**	**-2.585**	**14115**	**.001**

Note. Only significant clusters are depicted. Channel groups of significant main effects are specified in Supplementary Figure 3. Channels groups of the significant interaction are found in Figure 5 in the main text. Cluster *T*-statistics are evaluated (two-sided) for significance. A positive coefficient indicates higher GBA power in the test group than group mean. Effects with $p \leq .05$ are marked in bold. *P*-values of post-hoc tests are Holm corrected. The post-hoc tests are performed within the Group (rTLR) × *Emotion* interaction cluster. Abbreviations*:* CI = confidence interval (95 %), GBA = gamma-band activity, HC = healthy controls, lTLR = left temporal lobe resection, rTLR = right temporal lobe resection.

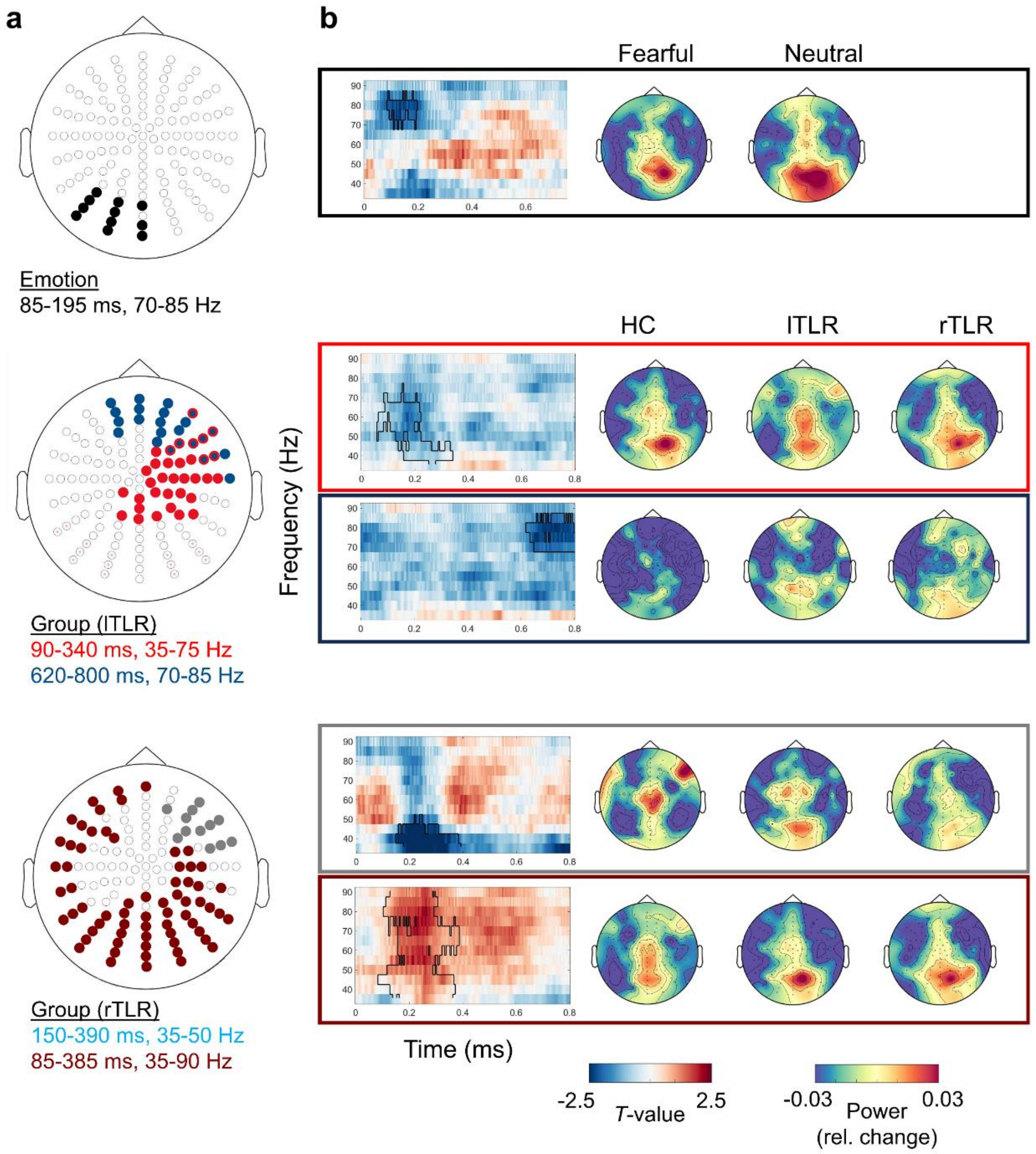

Supplementary Figure 3. Significant main effects in the GBA analysis. a Channel groups of significant clusters. Channels that were part of multiple clusters are colored respectively. **b** Time-frequency distribution of significant effects (left). Cluster bounds are marked with black outlines. Topographies (right) show grand-average GBA power, averaged across time- and frequency-points of the significant effect. Abbreviations: GBA = gamma-band activity, HC = healthy controls, lTLR = left temporal lobe resection, rTLR = right temporal lobe resection.

S3 Associations between behavior and electrophysiology

The models were calculated as follows:

$Behavior_{fearful\text{-}neutral} \sim P1_{fearful\text{-}neutral} * Group + N1_{fearful\text{-}neutral} * Group + EPN_{fearful\text{-}neutral} * Group + LPP_{fearful\text{-}neutral} * Group + GBA_{fearful\text{-}neutral} * Group + (1 \mid Group)$

Supplementary Table 9. Predictions of behavioral by electrophysiological emotion differentiation

Stimulus ratings	Arousal					Valence				
Coefficient	β	*SE*	*df*	*t*	*p*	β	*SE*	*df*	*t*	*p*
Intercept (Grand Mean)	**0.9340**	**0.275**	**35**	**3.421**	**.002**	**-0.638**	**0.202**	**35**	**-3.154**	**.003**
Group (lTLR)	-0.434	0.337	35	-1.288	.206	0.188	0.232	35	0.812	.422
Group (rTLR)	0.658	0.415	35	1.585	.122	0.113	0.303	35	0.373	.712
P1	-0.354	0.386	35	-0.915	.366	0.311	0.285	35	1.092	.282
N1	**-0.874**	**0.310**	**35**	**-2.818**	**.008**	0.347	0.213	35	1.626	.113
EPN	0.489	0.329	35	1.487	.146	-0.268	0.226	35	-1.184	.245
LPP	-0.539	0.357	35	-1.511	.140	-0.117	0.246	35	-0.476	.637
GBA	3.028	3.625	35	0.835	.409	0.042	2.494	35	0.017	.987
Group (lTLR) x P1	0.189	0.432	35	0.439	.664	-0.254	0.297	35	-0.854	.399
Group (rTLR) × P1	-0.333	0.558	35	-0.597	.555	0.435	0.384	35	1.133	.265
Group (lTLR) × N1	-0.203	0.390	35	-0.522	.605	0.083	0.268	35	0.309	.759
Group (rTLR) × N1	-0.018	0.473	35	-0.039	.969	0.155	0.326	35	0.477	.637
Group (lTLR) × EPN	0.822	0.435	35	1.891	.067	0.087	0.299	35	0.291	.773
Group (rTLR) × EPN	-0.074	0.445	35	-0.166	.869	-0.068	0.306	35	-0.222	.825
Group (lTLR) × LPP	0.130	0.484	35	0.268	.790	0.287	0.333	35	0.862	.395
Group (rTLR) × LPP	-0.172	0.569	35	-0.303	.764	-0.013	0.392	35	-0.032	.974
Group (lTLR) × GBA	6.208	5.669	35	1.095	.281	-2.506	3.901	35	-0.642	.525
Group (rTLR) × GBA	-5.896	4.756	35	-1.24	.223	5.520	3.273	35	1.686	.101
Recognition scores	DI					Bias				
Intercept (Grand Mean)	-0.092	0.060	35	-1.534	.134	**0.149**	**0.045**	**35**	**3.291**	**.002**
Group (lTLR)	-0.035	0.090	35	-0.387	.701	0.051	0.068	35	0.758	.453
Group (rTLR)	0.028	0.084	35	0.337	.738	-0.012	0.064	35	-0.187	.853
P1	0.092	0.073	35	1.256	.218	**-0.117**	**0.053**	**35**	**-2.215**	**.033**
N1	0.091	0.067	35	1.353	.185	0.068	0.049	35	1.409	.168
EPN	-0.103	0.071	35	-1.452	.155	-0.078	0.051	35	-1.525	.136
LPP	0.086	0.077	35	1.113	.273	-0.066	0.056	35	-1.178	.247
GBA	0.082	0.784	35	0.104	.918	-0.819	0.567	35	-1.444	.158
Group (lTLR) × P1	-0.064	0.093	35	-0.683	.499	0.026	0.068	35	0.385	.702
Group (rTLR) × P1	0.116	0.121	35	0.961	.343	-0.166	0.087	35	-1.898	.066
Group (lTLR) × N1	-0.151	0.084	35	-1.795	.081	-0.086	0.061	35	-1.403	.170
Group (rTLR) × N1	-0.036	0.102	35	-0.353	.726	0.012	0.074	35	0.163	.871
Group (lTLR) × EPN	0.182	0.094	35	1.937	.061	**0.182**	**0.068**	**35**	**2.679**	**.011**
Group (rTLR) × EPN	-0.010	0.096	35	-0.104	.918	-0.099	0.070	35	-1.421	.164
Group (lTLR) × LPP	0.080	0.105	35	0.767	.448	0.074	0.076	35	0.975	.336
Group (rTLR) × LPP	-0.049	0.123	35	-0.395	.695	**-0.201**	**0.089**	**35**	**-2.257**	**.030**
Group (lTLR) × GBA	1.171	1.226	35	0.955	.346	-0.591	0.888	35	-0.666	.510
Group (rTLR) × GBA	-0.480	1.029	35	-0.467	.644	0.841	0.745	35	1.13	.266

Note. A positive coefficient indicates higher amplitudes in the test condition than group mean. For factors with more than two levels, multiple contrasts were calculated. Effects with $p \leq .05$ are marked in bold. Abbreviations:

DI = Discrimination index, EPN = early posterior negativity, GBA = gamma-band activity, LPP = late positive potential, lTLR = left temporal lobe resection, rTLR = right temporal lobe resection, SE = standard error of mean.

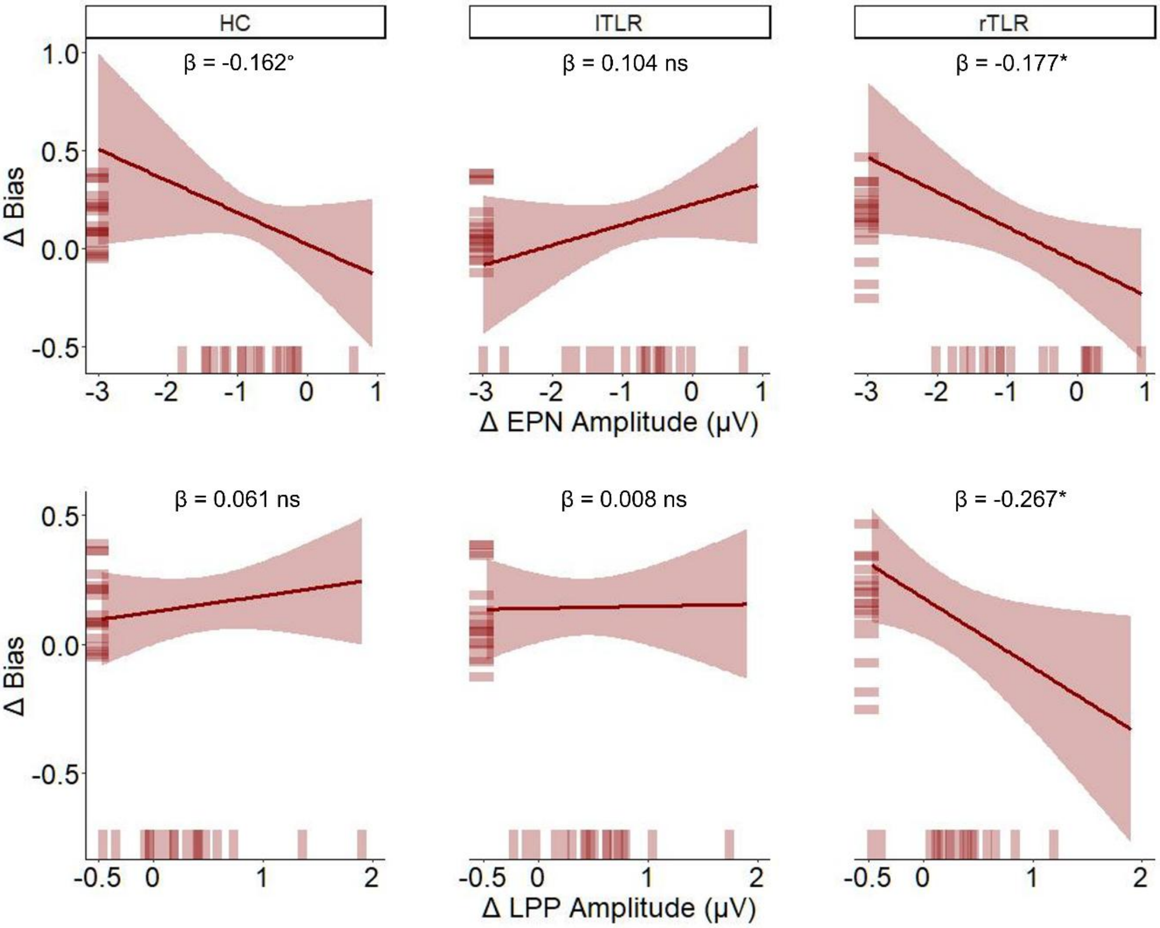

Supplementary Figure 4. Associations of differential ERP amplitudes and recognition bias. Lines depict average regression slopes (beta coefficients), shaded envelopes depict their standard error of measurement. Rug tassels show distribution of single difference values (fearful-neutral) for both the recognition bias and ERP component amplitudes (n = 18 per group). *$p \leq .05$, °$p \leq .10$. Abbreviations: EPN = early posterior negativity, HC = healthy controls, LPP = late positive potential, lTLR = left temporal lobe resection, ns = not significant, rTLR = right temporal lobe resection.

S4 Control analyses

Clinical variables and emotion differentiation

We conducted additional analyses to test whether emotion differentiation in both patient groups was modulated by clinical factors. First, we examined whether the resection volume of the fusiform gyrus (FG) and hippocampus differed between patient groups. These analyses revealed that FG resection volume was marginally larger for rTLR than lTLR patients ($t_{(22.585)} = 2.030$, $p = .054$). No differences were found for hippocampal volume ($t_{(32.267)} = 1.403$, $p = .170$). Then, we calculated the following control models:

ERP components.

$\text{Amplitude}_{\text{fearful-neutral}}$ ~ (Age at surgery + Months since resection + Age at epilepsy onset + BDI score + Ipsi-resectional hippocampal resection extent (in percentage) + Ipsi-resectional FG extent (in percentage)) * ERP component

Full model coefficients are documented in Supplementary Table 10.

GBA

$\text{Power}_{\text{fearful-neutral}}$ ~ Age at surgery + Months since resection + Age at epilepsy onset + BDI score + Ipsi-resectional hippocampal resection extent (in percentage) + Ipsi-resectional FG extent (in percentage)

Full model coefficients are documented in Supplementary Table 11.

Supplementary Table 10. Coefficients of the control models for the ERP components

	lTLR				rTLR			
Coefficient	β	*SE*	*t*	*p*	β	*SE*	*t*	*p*
Intercept (Grand Mean)	-0.311	0.432	-0.720	.475	**-0.671**	**0.279**	**-2.400**	**.021**
Age at surgery	-0.001	0.013	-0.101	.920	0.009	0.009	1.042	.303
Months since resection	-0.001	0.004	-0.142	.888	-0.008	0.004	-1.956	.057
Epilepsy onset	-0.002	0.012	-0.201	.841	-0.014	0.011	-1.262	.214
BDI	0.007	0.014	0.465	.644	0.007	0.015	0.475	.637
Hippocampus	-4.86e-04	0.009	-0.052	.959	0.011	0.008	1.502	.140
Fusiform	0.006	0.032	0.189	.851	0.013	0.008	1.670	.102
ERP (LPP)	0.981	0.747	1.313	.196	0.774	0.484	1.600	.117
ERP (N1)	-0.618	0.747	-0.826	.413	**-1.214**	**0.484**	**-2.508**	**.016**
ERP (P1)	0.065	0.747	0.086	.932	0.883	0.484	1.825	.075
Age at surgery × ERP (LPP)	0.009	0.023	0.372	.711	0.015	0.015	1.034	.307
Age at surgery × ERP (N1)	-0.008	0.023	-0.356	.724	-0.001	0.015	-0.041	.968
Age at surgery × ERP (P1)	0.001	0.023	0.043	.966	-0.008	0.015	-0.507	.615
Months since resection × ERP (LPP)	$1.23e^{-04}$	0.008	0.016	.987	0.008	0.007	1.160	.252
Months since resection × ERP (N1)	0.002	0.008	0.317	.753	-0.001	0.007	-0.140	.889
Months since resection × ERP (P1)	$3.68e^{-04}$	0.008	0.048	.962	-0.005	0.007	-0.755	.455
Epilepsy onset × ERP (LPP)	-0.015	0.021	-0.727	.471	0.004	0.019	0.214	.831
Epilepsy onset × ERP (N1)	0.018	0.021	0.874	.387	0.000	0.019	-0.018	.986
Epilepsy onset × ERP (P1)	-0.011	0.021	-0.508	.614	0.012	0.019	0.621	.538
BDI × ERP (LPP)	0.007	0.025	0.291	.772	-0.010	0.027	-0.390	.698
BDI × ERP (N1)	-0.012	0.025	-0.480	.634	0.003	0.027	0.118	.907
BDI × ERP (P1)	-0.007	0.025	-0.266	.792	-0.015	0.027	-0.544	.589
Hippocampus × ERP (LPP)	-0.001	0.016	-0.063	.950	-0.015	0.013	-1.118	.270
Hippocampus × ERP (N1)	0.007	0.016	0.464	.645	0.016	0.013	1.187	.242
Hippocampus × ERP (P1)	0.004	0.016	0.248	.806	-0.008	0.013	-0.600	.552
Fusiform × ERP (LPP)	-0.024	0.056	-0.425	.673	**-0.032**	**0.014**	**-2.304**	**.026**
Fusiform × ERP (N1)	-0.063	0.056	-1.124	.267	0.010	0.014	0.757	.453
Fusiform × ERP (P1)	0.064	0.056	1.134	.263	0.006	0.014	0.447	.657

Note. A positive coefficient indicates higher amplitudes in the test condition than group mean. The region-specific resection volume that was entered into the model only used ipsi-resectional volumes. Effects with $p \leq .05$ are marked in bold. Abbreviations: BDI = Beck's depression inventory, EPN = early posterior negativity, ERP = event-related potential, LPP = late positive potential, lTLR = left temporal lobe resection, rTLR = right temporal lobe resection, SE = standard error of mean.

Supplementary Table 11. Coefficients of the control models in GBA

	lTLR				rTLR			
Coefficient	β	*SE*	*t*	*p*	β	*SE*	*t*	*p*
Intercept (Grand Mean)	0.049	0.039	1.247	.238	-0.043	0.064	-0.674	.514
Age at surgery	-5.52e^{-04}	1.22e^{-03}	-0.454	.659	-4.50e^{-04}	1.97e^{-03}	-0.229	.823
Months since resection	-1.89e^{-04}	4.02e^{-04}	-0.470	.648	7.44e^{-04}	9.45e^{-04}	0.788	.448
Epilepsy onset	4.92e^{-04}	1.10e^{-03}	0.446	.664	-5.68e^{-04}	2.56e^{-03}	-0.222	.828
BDI	-4.04e^{-04}	1.30e^{-03}	-0.309	.763	1.57e^{-03}	3.51e^{-03}	0.447	.664
Hippocampus	-5.67e^{-04}	8.42e^{-04}	-0.673	.515	-4.12e^{-04}	1.72e^{-03}	-0.239	.815
Fusiform	-2.50e^{-04}	2.94e^{-03}	-0.085	.934	-1.12e^{-03}	1.82e^{-03}	-0.613	.553

Note. A positive coefficient indicates higher amplitudes in the test condition than group mean. The region-specific resection volume that was entered into the model only used ipsi-resectional volumes. Effects with $p \leq .05$ are marked in bold. Abbreviations*:* BDI = Beck's depression inventory, ERP = event-related potential, GBA = gamma-band activity, lTLR = left temporal lobe resection, rTLR = right temporal lobe resection, SE = standard error of mean.

SI **References**

1. Snodgrass, J. G. & Corwin, J. Pragmatics of measuring recognition memory: applications to dementia and amnesia. *Journal of Experimental Psychology: General* **117,** 34–50; 10.1037//0096-3445.117.1.34 (1988).
