# Supplementary material for: Fear perception as a function of hemisphere- and time-specific dynamics in the medial temporal lobes
Source: Commun Biol. 2025 Jul 25;8:1105. doi: 10.1038/s42003-025-08542-6 (PMC12297512; doi:10.1038/s42003-025-08542-6)
Supplement: Supplementary file 3 — Reporting Summary [file 42003_2025_8542_MOESM3_ESM.pdf]

nature portfolio

Corresponding author(s): Enya Marie Weidner

Last updated by author(s): May 28, 2025

Reporting Summary

Nature Portfolio wishes to improve the reproducibility of the work that we publish. This form provides structure for consistency and transparency in reporting. For further information on Nature Portfolio policies, see our Editorial Policies and the Editorial Policy Checklist.

Statistics

For all statistical analyses, confirm that the following items are present in the figure legend, table legend, main text, or Methods section.

n/a	Confirmed	
☐	☒	The exact sample size (*n*) for each experimental group/condition, given as a discrete number and unit of measurement
☐	☒	A statement on whether measurements were taken from distinct samples or whether the same sample was measured repeatedly
☐	☒	The statistical test(s) used AND whether they are one- or two-sided<br>*Only common tests should be described solely by name; describe more complex techniques in the Methods section.*
☐	☒	A description of all covariates tested
☐	☒	A description of any assumptions or corrections, such as tests of normality and adjustment for multiple comparisons
☐	☒	A full description of the statistical parameters including central tendency (e.g. means) or other basic estimates (e.g. regression coefficient) AND variation (e.g. standard deviation) or associated estimates of uncertainty (e.g. confidence intervals)
☐	☒	For null hypothesis testing, the test statistic (e.g. *F*, *t*, *r*) with confidence intervals, effect sizes, degrees of freedom and *P* value noted<br>*Give P values as exact values whenever suitable.*
☒	☐	For Bayesian analysis, information on the choice of priors and Markov chain Monte Carlo settings
☐	☒	For hierarchical and complex designs, identification of the appropriate level for tests and full reporting of outcomes
☐	☒	Estimates of effect sizes (e.g. Cohen's *d*, Pearson's *r*), indicating how they were calculated

Our web collection on statistics for biologists contains articles on many of the points above.

Software and code

Policy information about availability of computer code

Data collection	EEG was continuously recorded from 128 active BioSemi Ag-AgCl electrodes using the ActiView software (http://www.biosemi.com).
Data analysis	Custom code and the FieldTrip toolbox (version 20200121) in MATLAB (version 2019b, The MathWorks Inc.) were used to process EEG data and extract relevant measures. For linear mixed models (LMMs, lme4 v.1.1-34) on ERP data, data were exported to R running in RStudio (version 2023.03.1). LMMs on time-frequency data were calculated and evaluated in MATLAB.

For manuscripts utilizing custom algorithms or software that are central to the research but not yet described in published literature, software must be made available to editors and reviewers. We strongly encourage code deposition in a community repository (e.g. GitHub). See the Nature Portfolio guidelines for submitting code & software for further information.

Data

Policy information about availability of data

All manuscripts must include a data availability statement. This statement should provide the following information, where applicable:

- Accession codes, unique identifiers, or web links for publicly available datasets
- A description of any restrictions on data availability
- For clinical datasets or third party data, please ensure that the statement adheres to our policy

All data needed to evaluate the conclusions are present in the paper or the supporting information. The datasets and codes generated in this study are available

under this link: https://gitlab.ub.uni-bielefeld.de/ae02/weidnertlr so are numerical source data for graphs and charts. Raw data are available from the authors upon reasonable request to the corresponding author.

Research involving human participants, their data, or biological material

Policy information about studies with human participants or human data. See also policy information about sex, gender (identity/presentation), and sexual orientation and race, ethnicity and racism.

Reporting on sex and gender	Only information about biological sex was collected and reported based on self-reported data. Data from 18 rTLR patients (eight female), 18 lTLR patients (eight female), and 18 matched healthy controls (HC, nine female) are included in this report.
Reporting on race, ethnicity, or other socially relevant groupings	Participants were individually matched based on age (+/- 1 year), sex, and education (highest educational qualification) which were provided by the participants in a demographics questionnaire.
Population characteristics	Average age was 37.39 years (SD = 12.84) for the lTLR group, 34.06 years (SD = 11.93) for the rTLR group, and 35.17 years (SD = 12.13) for the HC group. For the patient groups, the average months since resection were 55.44 months (SD = 29.62) for the lTLR group and 48.06 months (SD = 20.44) for the rTLR group. Average age at epilepsy onset was 16.14 years (SD = 11.66) for the lTLR group and 15.61 years (SD = 8.87) for the rTLR group. Age at resection was, on average, 32.72 years (SD = 12.06) for the lTLR group and 30.06 years (SD = 11.36) for the rTLR group. Both patient groups showed higher BDI scores than controls (lTLR versus HC: t(20.322) = 2.281, P = .012; rTLR versus HC: t(25.099) = 2.965, P = .030), but only two fulfilled the criteria for a moderate or severe depressive episode. All rTLR patients and HC were right-handed, four lTLR patients were left-handed. Two of those showed typical left-sided language lateralization.
Recruitment	Patients were recruited through personal communication during routine checks in the hospital or via phone. Control subjects were recruited via personal communication, flyers, and online forums (Facebook). Informed consent of all participants was given prior to test days. A monetary reward of 100 Euros was given for participation in the entire study
Ethics oversight	The study was approved by the ethics committee of the German Psychological Association (DGPS).

Note that full information on the approval of the study protocol must also be provided in the manuscript.

Field-specific reporting

Please select the one below that is the best fit for your research. If you are not sure, read the appropriate sections before making your selection.

☒ Life sciences ☐ Behavioural & social sciences ☐ Ecological, evolutionary & environmental sciences

For a reference copy of the document with all sections, see nature.com/documents/nr-reporting-summary-flat.pdf

Life sciences study design

All studies must disclose on these points even when the disclosure is negative.

Sample size	No statistical methods were used to pre-determine sample sizes but our sample sizes are larger than those reported in previous publications (Framorando et al., 2021; Rotshtein et al., 2010).
Data exclusions	Data from 18 rTLR patients (eight female), 18 lTLR patients (eight female), and 18 matched healthy controls (HC, nine female) with no past or acute neurological and psychiatric disorders are included in this report. All groups were individually matched regarding age (+/- 1 year), sex, and education (highest educational qualification). Originally, 20 participants per group participated in this study. Two participants were excluded because of technical issues, three due to large perspiration and movement artifacts (n = 3), and one due to a large infarction in the left ventral visual pathway.
Replication	We performed control analyses excluding the 5 apical resections from the analysis. Analyses yielded the same results. Additionally, our rTLR results in the ERPs parallel data from image processing in the same sample reported elsewhere (Mielke et al., 2021).
Randomization	Grouping was pre-determined based on resection type. To avoid demographic confounds, groups were individually matched (see above). Emotion effects were evaluated based on within-subject analysis: all participants viewed all trials. The presentation of trials was randomized.
Blinding	As researchers who conducted data collection also recruited participants, complete blinding was not possible. However, instructions given to participants were highly standardized and researchers did not interact with participants during EEG recordings. Participants were not aware of the study's goal.

Reporting for specific materials, systems and methods

We require information from authors about some types of materials, experimental systems and methods used in many studies. Here, indicate whether each material, system or method listed is relevant to your study. If you are not sure if a list item applies to your research, read the appropriate section before selecting a response.

Materials & experimental systems

n/a	Involved in the study
☒	☐ Antibodies
☒	☐ Eukaryotic cell lines
☒	☐ Palaeontology and archaeology
☒	☐ Animals and other organisms
☒	☐ Clinical data
☒	☐ Dual use research of concern
☒	☐ Plants

Methods

n/a	Involved in the study
☒	☐ ChIP-seq
☒	☐ Flow cytometry
☐	☒ MRI-based neuroimaging

Plants

Seed stocks: /

Novel plant genotypes: /

Authentication: /

Magnetic resonance imaging

Experimental design

Design type: Clinical protocol for structural scanning

Design specifications: Structural scans were used for evaluation of resection extent

Behavioral performance measures: No behavioral measures were conducted during MRI scans.

Acquisition

Imaging type(s): structural MRI

Field strength: 3T Magnetom Verio Scanner (Siemens, Erlangen, Germany) with a 12-channel head coil

Sequence & imaging parameters: T1-weighted MRI

Area of acquisition: whole brain

Diffusion MRI: ☐ Used ☒ Not used

Preprocessing

Preprocessing software: SPM8(http://www.fil.ion.ucl.ac.uk/spm).

Normalization: Data were normalized to MNI space

Normalization template: MNI

Noise and artifact removal: As no functional data was analyzed, no noise and artifact removals were performed.

Volume censoring: Volume censoring was not used

Statistical modeling & inference

Model type and settings: No statistical models were calculated

Effect(s) tested: No effects were tested.

Specify type of analysis: ☒ Whole brain ☐ ROI-based ☐ Both

Statistic type for inference<br>(See Eklund et al. 2016)	Resected areas were traced manually in the individual structural T1 images of the patients and converted to Montreal Neurological Institute (MNI) space by applying the deformation fields derived from normalizing procedure in the preprocessing of the respective patient to the resection mask.
Correction	No corrections were performed.

Models & analysis

n/a	Involved in the study
☒	☐ Functional and/or effective connectivity
☒	☐ Graph analysis
☒	☐ Multivariate modeling or predictive analysis
